# A Phase Ib/II Randomized Clinical Trial of Oleclumab with or without Durvalumab plus Chemotherapy in Patients with Metastatic Pancreatic Ductal Adenocarcinoma

**DOI:** 10.1158/1078-0432.CCR-24-0499

**Published:** 2024-08-06

**Authors:** Andrew L. Coveler, Matthew J. Reilley, Mark Zalupski, Teresa Macarulla, Christos Fountzilas, Mariano Ponz-Sarvisé, Adnan Nagrial, Nataliya V. Uboha, Sophia Frentzas, Michael Overman, Anne Noonan, Wells A. Messersmith, Nick Pavlakis, Niharika B. Mettu, Ina Bisha, Ying Wang, Paul Smith, Elina Murtomaki, Agata A. Bielska, Veronique Bragulat, Zachary A. Cooper, Rakesh Kumar, David R. Spigel

**Affiliations:** 1 Fred Hutchinson Cancer Center, Seattle, Washington.; 2 University of Virginia Comprehensive Cancer Center, Charlottesville, Virginia.; 3 University of Michigan Health System, Ann Arbor, Michigan.; 4 Vall d’Hebrón Institute of Oncology (VHIO), Vall d’Hebrón University Hospital, Barcelona, Spain.; 5 Roswell Park Comprehensive Cancer Center, Buffalo, New York.; 6 Cancer Center Clinica Universidad de Navarra, Pamplona, Spain.; 7 Blacktown Hospital, Sydney, Australia.; 8 University of Wisconsin Carbone Cancer Center, Madison, Wisconsin.; 9 Monash Medical Centre, Clayton, Australia.; 10 The University of Texas MD Anderson Cancer Center, Houston, Texas.; 11 The Ohio State University Comprehensive Cancer Center, Columbus, Ohio.; 12 University of Colorado Cancer Center, Aurora, Colorado.; 13 Northern Sydney Cancer Centre, Royal North Shore Hospital, Sydney, Australia.; 14 Duke University Medical Center, Durham, North Carolina.; 15 AstraZeneca, Munich, Germany.; 16 AstraZeneca, Waltham, Massachusetts.; 17 On behalf of AstraZeneca, Cambridge, United Kingdom.; 18 AstraZeneca, Cambridge, United Kingdom.; 19 AstraZeneca, Gaithersburg, Maryland.; 20 Sarah Cannon Research Institute, Nashville, Tennessee.

## Abstract

**Purpose:**

Pancreatic ductal adenocarcinoma upregulates CD73, potentially contributing to immune surveillance evasion. Combining oleclumab (CD73 inhibitor) and durvalumab with chemotherapy may identify an effective treatment option.

**Patients and Methods:**

We describe a multicenter phase Ib/II randomized clinical trial in patients with metastatic pancreatic ductal adenocarcinoma, untreated (cohort A) or previously received gemcitabine-based chemotherapy (cohort B; NCT03611556). During escalation, patients received oleclumab 1,500 or 3,000 mg, durvalumab 1,500 mg, and gemcitabine plus nab-paclitaxel (GnP; cohort A; *n* = 14) or modified FOLFOX (cohort B; *n* = 11). During expansion, cohort A patients (*n* = 170) were randomized to GnP (arm A1), oleclumab [recommended phase II dose (RP2D)] with GnP (arm A2), or oleclumab (RP2D) with durvalumab plus GnP (arm A3). Primary objectives were safety (escalation) and objective response rate (expansion). Secondary objectives included progression-free survival (PFS) and overall survival (OS).

**Results:**

During escalation, 1/11 patients from cohort B (oleclumab 3,000 mg) experienced two dose-limiting toxicities. Oleclumab’s RP2D was 3,000 mg. During expansion, grade ≥3 treatment-related adverse events occurred in 67.7% (42/62) of patients in A1, 73.7% (28/38) in A2, and 77.1% (54/70) in A3. The objective response rate was 29.0%, 21.1%, and 32.9% in A1, A2, and A3, respectively (A1 vs. A3; *P* = 0.650). PFS [HR = 0.72; 95% confidence interval (CI), 0.47, 1.11] and OS (HR = 0.75; 95% CI, 0.50–1.13) were similar for A3 versus A1. Patients with high CD73 expression had improved PFS and OS in A3 versus A1, although this should be interpreted with caution.

**Conclusions:**

Although the safety profile was acceptable, this study did not meet its primary efficacy endpoint.

**Translational Relevance**

Upregulation of CD73 is thought to help pancreatic ductal adenocarcinoma (PDAC) cells evade immune surveillance, and increased CD73 expression is associated with poorer outcomes in patients with PDAC. Targeting CD73 may therefore be an effective treatment option for the management of PDAC. In this randomized phase Ib/II dose-escalation and dose-expansion study of oleclumab (anti–CD73 human mAb) and gemcitabine plus nab-paclitaxel chemotherapy with or without durvalumab in patients with metastatic PDAC, safety and tolerability were acceptable. The primary efficacy endpoint of an increased objective response rate with oleclumab with/without durvalumab plus chemotherapy versus chemotherapy alone was not met; therefore, any future studies should consider biomarker-based selection.

## Introduction

Pancreatic ductal adenocarcinoma (PDAC) has an extremely poor prognosis, with a 5-year relative survival rate of 13% in the United States (1). Currently, recommended treatment for metastatic PDAC (mPDAC) generally involves combination chemotherapy (2, 3); however, the effectiveness of these regimens remains limited. PD(L)1 inhibitors have demonstrated efficacy in various solid tumors, although not in pancreatic cancer (4); however, chemotherapy used in patients with pancreatic cancer may promote immunogenic cell death, and preclinical studies and preliminary clinical reports provide evidence for the rationale to combine chemotherapy with PD(L)1 inhibitors in these patients (2). Cluster of differentiation 73 (CD73) is expressed on the surface of cancer cells, fibroblasts, and immune cells (5, 6) and converts AMP to adenosine, which is immunosuppressive in the tumor microenvironment (7, 8). Numerous tumor types, including PDAC, upregulate CD73 to evade immune surveillance (9–13). Elevated tumor CD73 expression is associated with poorer outcomes in several cancers, including PDAC (13–16). Targeting CD73 may overcome resistance to immunotherapy and improve treatment outcomes through combinatorial approaches that stimulate antitumor immune activity and potentially sensitize or resensitize tumors to chemotherapy and immunotherapy (10–12).

Oleclumab is a high-affinity (dissociation constant: 0.113 nmol/L) human IgG1λ mAb that potently and selectively inhibits the catalytic activity of CD73 via steric blocking and inter-CD73 dimer cross-linking and decreases CD73 expression through internalization, thereby inhibiting the production of immunosuppressive extracellular adenosine (7, 8, 17). In the first-in-human study of oleclumab, CD73 enzymatic activity was decreased in all four patients with pre- and posttreatment tissue samples 20 days after the first oleclumab dose (18). Additionally, a sustained decrease in free soluble CD73 and CD73 on peripheral T lymphocytes was observed. In this phase I study, oleclumab in combination with durvalumab showed antitumor activity in patients with previously treated advanced PDAC (18). Oleclumab monotherapy was associated with treatment-related adverse events (TRAE) in 55% of patients, most commonly fatigue (17%) and nausea (10%); grade 3 to 4 TRAEs were observed in 7% of patients (18). In patients with unresectable stage III non-small cell lung cancer and no progression after concurrent chemoradiotherapy, the combination of oleclumab with durvalumab (PDL1 inhibitor) demonstrated improved objective response rate [ORR; 30.0% (95% confidence interval (CI), 18.8, 43.2) vs. 17.9 (95% CI, 9.6, 29.2)] and progression-free survival [PFS; HR = 0.44; 95% CI, 0.26, 0.75] compared with durvalumab alone, with no additional safety signals reported (19). Here, we describe a phase Ib/II study of oleclumab combined with durvalumab and chemotherapy in patients with mPDAC.

## Patients and Methods

### Study design

This multicenter, open-label, dose-escalation, and dose-expansion study included patients who were previously untreated (cohort A), and patients who had previously received gemcitabine-based chemotherapy without exposure to 5-fluorouracil, capecitabine, or oxaliplatin (cohort B; Supplementary Fig. S1).

The primary objective of the dose-escalation phase was to assess safety and tolerability; objectives and endpoints of the dose-escalation phase are detailed fully in Supplementary Methods. The primary objective of the dose-expansion phase was to evaluate preliminary antitumor activity. The primary endpoint was investigator-assessed ORR per RECIST v1.1 criteria. Secondary objectives were further evaluation of antitumor activity, including assessments by baseline CD73 level, safety, and tolerability, and evaluation of pharmacokinetics (PK) and immunogenicity. Secondary endpoints included PFS, duration of response (DoR), and disease control rate per RECIST v1.1 and overall survival (OS), incidence of adverse events (AE), and serious AEs (SAE), clinically meaningful changes from baseline in laboratory parameters and vital signs, summary PK, and development of detectable antidrug antibodies (ADA). Exploratory objectives included monitoring of potential biomarkers for correlation with biological activity or identification of patients most likely to benefit from treatment. Exploratory endpoints included assessment of mutational phenotypes and protein expression levels. There was no crossover between treatment arms.

### Treatments

During dose-escalation, all patients received i.v. oleclumab 1,500 or 3,000 mg every 2 weeks (days 1 and 15 of each 28-day treatment cycle) for four doses, then once every 4 weeks (Q4W), in combination with i.v. durvalumab 1,500 mg Q4W (day 1 of each treatment cycle).

Patients in cohort A received systemic chemotherapy with i.v. gemcitabine (1,000 mg/m^2^) and nab-paclitaxel (125 mg/m^2^; GnP) on days 1, 8, and 15 of each 28-day treatment cycle; patients in cohort B received chemotherapy with modified (m)FOLFOX (oxaliplatin 85 mg/m^2^ i.v., leucovorin 400 mg/m^2^ i.v., and 5-fluorouracil 400 mg/m^2^ i.v. bolus followed by 2,400 mg/m^2^ continuous infusion over 46–48 hours) on days 1 and 15 of each 28-day treatment cycle.

During dose-expansion, patients in cohort A were randomized 1:1:1 via interactive response technology, and stratified by high or low CD73 expression, to either GnP alone per the schedule in dose-escalation (arm A1), oleclumab [at the recommended phase II dose (RP2D)] plus GnP (arm A2), or oleclumab (at the RP2D) with durvalumab plus GnP (arm A3) per the schedule in dose-escalation. Cohort B of the dose-expansion phase was not opened.

All patients were treated until disease progression, intolerable toxicity, withdrawal of consent, fulfillment of another discontinuation criterion, or study closure. Access to the study therapy after study closure was provided for patients who, according to the investigator, gained clinical benefit from extended access to the study drug.

### Patients

Patients were aged ≥18 years and had histologically/cytologically confirmed mPDAC with ≥1 measurable lesion per RECIST v1.1, an Eastern Cooperative Oncology Group performance score of 0 or 1, and adequate organ and marrow function. Patients were excluded if they had received prior immune-related therapy including agents targeting PDL1, CTLA4, CD73, CD39, or adenosine receptors (except therapeutic anticancer vaccines); if they had any unresolved toxicity (excluding alopecia) from prior standard therapy; or if they experienced venous thrombosis, myocardial infarction, transient ischemic attack, or stroke within the past 3 months (see Supplementary Methods for other inclusion/exclusion criteria).

All patients provided written informed consent to participate in the study, including consent to provide an archival or fresh tumor specimen for correlative biomarker and CD73 expression testing for the dose-expansion phase randomization stratification. The study protocol was approved by the institutional review board or ethics committee for each participating center, and the study was run in accordance with ethical principles originating in the Declaration of Helsinki and consistent with the International Conference on Harmonization guidelines on Good Clinical Practice, as well as applicable regulatory requirements.

### Assessments

Safety in both phases was monitored by physical examination, ECG, laboratory testing, vital signs, and AEs/SAEs graded according to NCI Common Terminology Criteria for Adverse Events version 4.03. Dose-limiting toxicities (DLT), defined as any ≥grade 3 toxicities, were evaluated during dose escalation from the first dose of all study treatments through day 28 (see Supplementary Methods for further DLT definition).

Disease assessments per RECIST v1.1 were performed as described (see Supplementary Methods). Response was assessed using CT with contrast or MRI of the chest, abdomen, and pelvis, and MRI of the brain if indicated.

During dose expansion, patients were stratified by high or low tumoral expression of CD73, determined in a College of American Pathologists/Clinical Laboratory Improvement Amendments laboratory using a validated IHC assay. High and low CD73 expression levels were defined as 2+ or 3+ intensity in ≥50% of tumor cells or <50% of tumor cells, respectively.

Details on IHC and analyses of next-generation sequencing data from ctDNA can be found in Supplementary Methods.

### Statistical considerations

#### Planned number of patients and interim analysis

For cohort A of the dose-expansion phase, a sample size of 70 patients per arm was planned, based on an ORR with GnP (arm A1) of 23% (26) and an assumed dropout rate of 20% providing 78% power at a one-sided significance level of 0.10 to detect a difference in ORR of 20% [i.e., ORR = 43% in the oleclumab-containing arms (arms A2 or A3; see Supplementary Methods)]. Randomization was paused for interim futility analysis (see Supplementary Methods) when ∼30 patients with a baseline disease assessment in each arm had been dosed for at least 16 weeks and had at least one postbaseline disease assessment and/or discontinued treatment due to disease progression or death.

#### Safety

Descriptive summaries of safety parameters, including DLTs, AEs, SAEs, laboratory evaluations, vital signs, and ECG results, were based on the as-treated population (see Supplementary Methods for description of study populations).

#### PK and immunogenicity

Only patients who received at least one dose of oleclumab and/or durvalumab with at least one reportable sample were evaluated. For each cohort, the immunogenic potential of combinations was assessed by summarizing the number and percentage of patients who developed detectable ADAs. Individual oleclumab and durvalumab concentrations were tabulated by dose cohort along with descriptive statistics. Noncompartmental PK parameters were not calculated as sparse PK samples were collected.

#### Efficacy

Analyses of antitumor activity during dose-escalation were based on the as-treated population (see Supplementary Methods for description of study populations); analyses during dose-expansion were conducted on the intention-to-treat population. ORR and disease control rate per RECIST v1.1 were summarized with 95% CIs based on exact binomial distribution. Comparison of arms for ORR was obtained via Cochran-Mantel-Haenszel testing (stratified by CD73 level) in order to detect a difference in ORR of 20%. Time-to-event endpoints (DoR, PFS, and OS) were analyzed by Kaplan–Meier methodology. Comparison of PFS and OS across arms was obtained using the log-rank test with the HR and 95% CI estimated by Cox regression, both of which were stratified by CD73 level.

Analysis of ORR, PFS, and OS by CD73 level at baseline was also performed via the Fisher exact test (for ORR analysis) and Cox proportional-hazards model (for PFS and OS).

### Data availability

Data underlying the findings described in this article may be obtained in accordance with AstraZeneca’s data sharing policy described at https://astrazenecagrouptrials.pharmacm.com/ST/Submission/Disclosure. Data for studies directly listed on Vivli can be requested through Vivli at www.vivli.org. Data for studies not listed on Vivli could be requested through Vivli at https://vivli.org/members/enquiries-about-studies-not-listed-on-the-vivli-platform/. AstraZeneca’s Vivli member page is also available outlining further details: https://vivli.org/ourmember/astrazeneca/.

## Results

At the data cutoff date of July 22, 2022, 195 patients, including 25 in the dose-escalation phase and 170 in the dose-expansion phase, received treatment across 29 sites from four countries.

### Dose-escalation phase

Results from the dose-escalation phase are presented in the Supplementary Results and in Supplementary Tables S1–S3. Oleclumab 3,000 mg was chosen as the RP2D for dose-expansion.

### Dose-expansion phase

#### Patient demographics and disease characteristics

In dose-expansion cohort A, 170 patients were randomized and received first-line treatment with either GnP (arm A1, *n* = 62), oleclumab plus GnP (arm A2, *n* = 38), or oleclumab with durvalumab plus GnP (arm A3, *n* = 70). Patient demographics were generally well balanced across the treatment arms (Table 1). Disease characteristics were generally similar except for the median CA19-9 value which was higher in arm A3 than in arms A1 and A2. The patients included in this study were generally representative of the disease population (Supplementary Table S4)

**Table 1.** Patient demographics and disease characteristics in the dose-expansion phase (intent-to-treat population; *N* = 170).

Characteristic	Arm A1, 1L GnP (*N* = 62)	Arm A2, 1L O + GnP (*N* = 38)	Arm A3, 1L O + D + GnP (*N* = 70)
Median age (range), years	66.0 (49, 85)	61.0 (30, 80)	62.5 (39, 78)
Sex, *n* (%)			
Female	26 (41.9)	17 (44.7)	34 (48.6)
Male	36 (58.1)	21 (55.3)	36 (51.4)
Race, *n* (%)			
American Indian or Alaska Native	0	0	1 (1.4)
Asian	5 (8.1)	1 (2.6)	1 (1.4)
Black or African American	3 (4.8)	2 (5.3)	2 (2.9)
Native Hawaiian or other Pacific Islander	0	0	0
White	54 (87.1)	33 (86.8)	64 (91.4)
Other	0	1 (2.6)	2 (2.9)
≥2 races	0	1 (2.6)	0
ECOG PS, *n* (%)			
0	26 (41.9)	20 (52.6)	33 (47.1)
1	36 (58.1)	18 (47.4)	37 (52.9)
Time since initial diagnosis, median (range), months	1.2 (1, 79)	1.3 (0, 149)	1.2 (1, 49)
Baseline CA19-9^a^, median (range), U/mL	1475.4 (1, 9518270)	1067.3 (0, 457080)	2957.5 (2, 3465000)
Liver metastasis, *n* (%)			
Yes	47 (75.8)	26 (68.4)	54 (77.1)
No/not specified	15 (24.2)	12 (31.6)	16 (22.9)
Tumor CD73 expression, *n* (%)^b^			
Low	16 (26)	11 (29)	19 (27)
High	46 (74)	27 (71)	51 (73)
PDL1 expression ≥1%, *n* (%)^c^			
Tumor cells	13 (23.2)	15 (46.9)	20 (29.0)
Immune cells	35 (62.5)	23 (71.9)	35 (50.7)

Abbreviations: D, durvalumab; ECOG PS, Eastern Cooperative Oncology Group performance status; 1L, first line; O, oleclumab.

a Assessed by a local laboratory.

b CD73 low: no CD73 expression in tumor cells or <50% of tumor cells with 2+ or 3+ intensity; CD73 high: CD73 expression of 2+ or 3+ intensity in ≥50% of tumor cells.

c Dose-expansion arm A1, *n* = 56; arm A2, *n* = 32; arm A3, *n* = 69.

**Figure 1.**
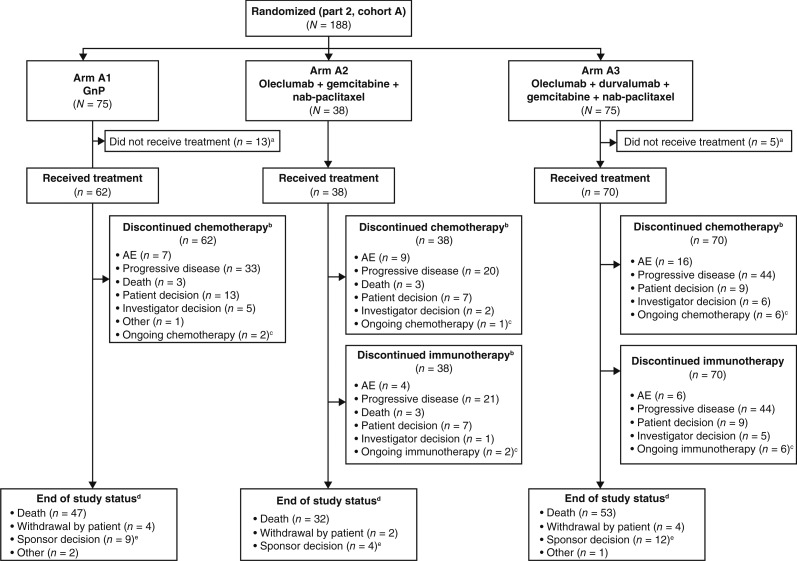
CONSORT diagram for the dose-expansion phase. “a” denotes arm A1: death (*n* = 1); withdrawal by patient (*n* = 8); other (*n* = 4). Arm A3: death (*n* = 1); withdrawal by patient (*n* = 2); other (*n* = 2). “b” denotes patients could be counted more than once in this category if they discontinued different chemotherapy options for different reasons. “c” denotes as permitted per study protocol, patients from arms A2 and A3 continued to receive immunotherapy off-study after the study closure on July 22, 2022, and patients from all arms continued to receive treatment per standard of care at their site. “d” denotes end of study status as defined in the protocol. “e” denotes “sponsor decision" and includes patients either still on treatment or in survival follow-up at the time of study closure. *N*, number of patients in each treatment group.

Patient disposition is summarized in Fig. 1. Fewer patients were treated in arm A2 than arms A1 and A3 due to a sponsor decision to halt recruitment into arm A2 after interim efficacy analyses (see Supplementary Methods) and to expand recruitment as planned into arms A1 and A3; thus, the focus of the results is on the comparison of arms A3 and A1. Disposition characteristics were generally similar across all treatment arms.

#### Safety

The median number of oleclumab doses was 6.5 in arm A2 (range 1–41) and 8.0 in arm A3 (range 1–32). The overall safety profile in arms A2 and A3 was consistent with the known safety profiles of each component, and no new safety signals were identified (Table 2).

**Table 2.** TEAEs occurring at any severity in ≥20% of patients in any arm in the dose-expansion phase (as-treated population; *N* = 170).

TEAE, *n* (%)	Arm A1, 1L GnP (*N* = 62)		Arm A2, 1L O + GnP (*N* = 38)		Arm A3, 1L O + D + GnP (*N* = 70)	
	Any grade	Grade ≥3	Any grade	Grade ≥3	Any grade	Grade ≥3
Any TEAE	62 (100)	53 (85.5)	37 (97.4)	34 (89.5)	70 (100)	63 (90.0)
TEAEs occurring in >20% of patients in any cohort^a^						
Fatigue	30 (48.4)	8 (12.9)	25 (65.8)	4 (10.5)	41 (58.6)	5 (7.1)
Nausea	29 (46.8)	5 (8.1)	26 (68.4)	1 (2.6)	41 (58.6)	1 (1.4)
Diarrhea	20 (32.3)	3 (4.8)	14 (36.8)	2 (5.3)	38 (54.3)	4 (5.7)
Anemia	17 (27.4)	8 (12.9)	14 (36.8)	3 (7.9)	28 (40.0)	11 (15.7)
Neutropenia	22 (35.5)	14 (22.6)	15 (39.5)	13 (34.2)	16 (22.9)	12 (17.1)
Peripheral edema	13 (21.0)	1 (1.6)	12 (31.6)	2 (5.3)	28 (40.0)	0
Decreased appetite	16 (25.8)	2 (3.2)	15 (39.5)	0	21 (30.0)	1 (1.4)
Neutrophil count decreased	19 (30.6)	16 (25.8)	8 (21.1)	5 (13.2)	24 (34.3)	16 (22.9)
Vomiting	20 (32.3)	4 (6.5)	12 (31.6)	0	17 (24.3)	3 (4.3)
Alopecia	12 (19.4)	0	11 (28.9)	0	25 (35.7)	0
Pyrexia	15 (24.2)	3 (4.8)	12 (31.6)	1 (2.6)	21 (30.0)	3 (4.3)
Constipation	10 (16.1)	0	16 (42.1)	1 (2.6)	19 (27.1)	0
Platelet count decreased	13 (21.0)	0	9 (23.7)	4 (10.5)	20 (28.6)	8 (11.4)
Abdominal pain	14 (22.6)	5 (8.1)	6 (15.8)	1 (2.6)	15 (21.4)	3 (4.3)
Peripheral neuropathy	11 (17.7)	1 (1.6)	11 (28.9)	0	13 (18.6)	0
Peripheral sensory neuropathy	9 (14.5)	1 (1.6)	11 (28.9)	0	15 (21.4)	0
AST increased	11 (17.7)	3 (4.8)	8 (21.1)	3 (7.9)	15 (21.4)	5 (7.1)
ALT increased	8 (12.9)	1 (1.6)	9 (23.7)	6 (15.8)	16 (22.9)	7 (10.0)
Pruritus	5 (8.1)	0	6 (15.8)	0	16 (22.9)	0
Myalgia	3 (4.8)	0	8 (21.1)	0	14 (20.0)	0
Rash	4 (6.5)	0	6 (15.8)	0	14 (20.0)	3 (4.3)

Abbreviations: ALT, alanine aminotransferase, AST aspartate aminotransferase, D, durvalumab; 1L, first line; MedDRA, Medical Dictionary for Regulatory Activities; O, oleclumab.

MedDRA version: 25.0.

a TEAEs listed in order of total overall frequency across all cohorts. Patients are counted once for each System Organ Class and Preferred Term regardless of the number of events.

The most common treatment-emergent AEs (TEAE; ≥40% total patients) were nausea (46.8% in arm A1, 68.4% in arm A2, and 58.6% in arm A3), fatigue (48.4% in arm A1, 65.8% in arm A2, and 58.6% in arm A3), and diarrhea (32.3% in arm A1, 36.8% in arm A2, and 54.3% in arm A3; Table 2). The rates of TEAEs leading to discontinuation were higher in arms A3 (24.3%) and A2 (23.7%) than in arm A1 (11.3%; Supplementary Table S5); TEAEs of special interest (AESI) for oleclumab occurred in 65.8% of patients in arm A2 and 61.4% in arm A3 versus 37.1% in arm A1. The most commonly reported AESI categories (≥20% total patients) were microvascular capillary permeability (25.8% in arm A1, 42.1% in arm A2, and 48.6% in arm A3) and thromboembolic events (19.4% in arm A1, 23.7% in arm A2, and 24.3% in arm A3). Although microvascular capillary permeability rates were higher in arms A2 and A3, this was driven by grade 1 and 2 events. Rates of grade 3 microvascular capillary permeability were similar across treatment arms (3.2% in arm A1, 5.3% in arm A2, and 5.7% in arm A3), with no reported grade 4 or 5 events in this category. For durvalumab, AESI occurred in 94.3% of patients in arm A3 versus 64.5% in arm A1 and 81.6% in arm A2. The most commonly reported AESI categories were diarrhea/colitis (37.1% in arm A1, 39.5% in arm A2, and 55.7% in arm A3), dermatitis/rash (25.8% in arm A1, 44.7% in arm A2, and 48.6% in arm A3), and hepatic events (21.0% in arm A1, 34.2% in arm A2, and 31.4% in arm A3).

TRAEs occurred in nearly all patients and were of grade ≥3 in 67.7% of patients in arm A1, 73.7% in arm A2, and 77.1% in arm A3 (Supplementary Table S5). The most common grade ≥3 TRAEs were primarily hematologic (Supplementary Table S6). The rates of serious TRAEs were similar across treatment arms; 19.4% in arm A1, 23.7% in arm A2, and 24.3% in arm A3. The most common serious TRAEs were pyrexia (4.8% in arm A1, 7.9% in arm A2, and 8.6% in arm A3), pneumonitis (1.6% in arm A1, 0 in arm A2, and 5.7% in arm A3), and diarrhea (1.6% in arm A1, 2.6% in arm A2, and 2.9% in arm A3). Two patients (1.2%) died because of TRAEs: one in arm A1 with infectious enterocolitis and lung infection/pneumonia related to chemotherapy and the other in arm A3 with intracranial hemorrhage related to oleclumab and durvalumab.

#### PK and immunogenicity

Oleclumab mean serum concentrations are shown in Supplementary Table S7. The PK of oleclumab were similar to a previous study (18). Additionally, there were no marked differences in oleclumab serum concentrations between arm A2 and arm A3, indicating that the combination with durvalumab did not affect the PK of oleclumab.

Immunogenicity of oleclumab was low in arms A2 and A3. None of the 94 evaluable patients had ADAs at baseline, and one patient had ADAs postbaseline.

#### Efficacy

**Figure 2.**
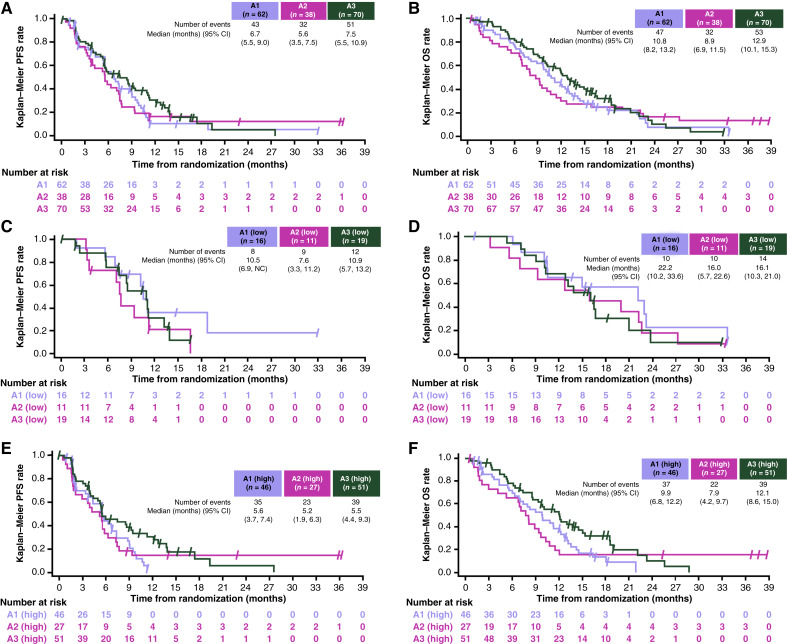
**A,** PFS and (**B**) OS in the dose-expansion phase overall (ITT) population; (**C**) PFS and (**D**) OS in patients with low CD73 levels, and (**E**) PFS and (**F**) OS in patients with high CD73 levels. ITT, intent-to-treat; NC, not calculable.

The dose-expansion part of the study did not meet its primary endpoint to demonstrate a statistically significant improvement in ORR when comparing oleclumab with or without durvalumab in combination with GnP versus GnP alone (Table 3). ORR was similar in arm A3 (32.9%) and arm A1 (29.0%; ORR difference: 3.8%, 95% CI, −13.2, 20.7; *P* = 0.650). ORR in arm A2 was 21.1%. All responses were partial responses, with DoR ranging from 1.8 to 30.5 months. The median DoR was numerically longer for arms A2 and A3 than arm A1. ORR was also similar in the CD73-high population: 31.4% in arm A3 and 23.9% in arm A1 (ORR difference: 7.5%; 95% CI, −12.6, 27) and 22.2% in arm A2 (Supplementary Table S8). There was no significant improvement in PFS (HR = 0.72; 95% CI, 0.47, 1.11) and OS (HR = 0.75; 95% CI, 0.50, 1.13) in the overall population in arm A3 (median PFS, 7.5 months; median OS, 12.9 months) compared with arm A1 (median PFS, 6.7 months; median OS, 10.8 months; Table 3; Fig. 2). The median PFS (5.6 months) and OS (8.9 months) were numerically shorter in arm A2 than in arm A1. In the CD73-high population, patients in arm A3 had improved PFS (HR = 0.60; 95% CI, 0.37, 0.97) and OS (HR = 0.61; 95% CI, 0.38, 0.97) compared with patients in arm A1 (median PFS, 5.5 vs. 5.6 months and median OS, 12.1 vs. 9.9 months for arm A3 vs. arm A1, respectively; Fig. 2; Supplementary Table S8). As was observed for the total population, the median PFS (5.2 months) and OS (7.9 months) in the CD73-high population were numerically shorter in arm A2 than in arm A1.

**Table 3.** Efficacy in the dose-expansion phase (intent-to-treat population; *N* = 170).

	Arm A1, 1L GnP (*N* = 62)	Arm A2, 1L O + GnP (*N* = 38)	Arm A3, 1L O + D + GnP (*N* = 70)
ORR, % (95% CI)	29.0 (18.2–41.9)	21.1 (9.6–37.3)	32.9 (22.1–45.1)
ORR difference, % (95% CI)	—	−8.0 (−27.6 to 12.1)	3.8 (−13.2 to 20.7)
*P* value for difference^a^		0.361	0.650
Best response, *n* (%)			
Partial response	18 (29.0)	8 (21.1)	23 (32.9)
Stable disease	26 (41.9)	20 (52.6)	30 (42.9)
Progressive disease	8 (12.9)	3 (7.9)	11 (15.7)
Median DoR, months (range)	7.2 (1.8–14.7)	12.9 (2.2–30.5)	9.5 (1.9–16.5)
Disease control^b^, *n* (%)	41 (66.1)	28 (73.7)	53 (75.7)
Median PFS, months (95% CI)	6.7 (5.5–9.0)	5.6 (3.5–7.5)	7.5 (5.5–10.9)
PFS HR (95% CI)	—	1.16 (0.73–1.84)	0.72 (0.47–1.11)
Median OS, months (95% CI)	10.8 (8.2–13.2)	8.9 (6.9–11.5)	12.9 (10.1–15.3)
OS HR (95% CI)	—	1.26 (0.79–1.98)	0.75 (0.50–1.13)

Abbreviations: D, durvalumab; 1L, first line; O, oleclumab.

*P* values and HRs are for treatment comparisons to arm A1 (1L GnP). An HR <1 favors arm A3/arm A2 (as applicable).

a *P* value for comparison of treatment groups obtained from the Cochran–Mantel–Haenszel test stratified by CD73 level.

b Disease control; complete responses plus partial responses plus stable disease ≥8 weeks.

#### Biomarker analysis

Exploratory IHC analyses of the predictive value of tumoral PDL1, CD8, or CD39 demonstrated no additional OS benefit when comparing arm A3 with arm A1 (Supplementary Fig. S2A). In the CD73-high population, OS benefit was observed in the CD8-high population, PDL1 tumor cell–negative population, and patients with low tumor mutational burden (TMB), *KRAS* mutations, or without homologous recombination repair mutations when comparing arm A3 with arm A1 (Supplementary Fig. S2B).

Additionally, baseline plasma samples (*n* = 160) were assessed for ctDNA, mutations, and blood TMB scores for each patient (see Supplementary Methods). *KRAS* and *TP53* mutations were the most prominent somatic mutations observed in 73% and 71% of the patients, respectively (Supplementary Fig. S3). Higher CD73 expression was observed in patients who had *KRAS* mutations (Supplementary Fig. S4A). Similar to a previous study (20), *KRAS* mutations trended toward a shorter OS and PFS across arms (Supplementary Fig. S4B). Furthermore, high baseline median variant allelic frequency in ctDNA was enriched with *KRAS*-mutant tumors and was associated with shorter OS and PFS in arm 3 (Supplementary Fig. S5A and S5B). The distributions of patients with *KRAS* mutations, median variant allele fraction, and TMB were similar across arms (Supplementary Fig. S5C–S5E).

## Discussion

In this study, the addition of oleclumab and durvalumab to standard-of-care chemotherapy showed a manageable safety profile in patients with mPDAC in the cohort A (GnP) and B (mFOLFOX) dose-escalation phase and in the cohort A dose-expansion phase. There were no unexpected TEAEs and TRAEs, and the overall safety profile of oleclumab plus durvalumab with GnP was consistent with their known individual safety profiles. Microvascular capillary permeability is an AESI for oleclumab due to its mechanism of action. In the present study, the rate of microvascular capillary permeability was high across all treatment arms; this high basal rate may reflect both the patient population and underlying disease, as well as the chemotherapy used across all treatment arms. The increased rates of microvascular capillary permeability observed in the treatment arms containing oleclumab were driven by grade 1 and 2 events.

The study did not meet its primary efficacy endpoint of an increase in ORR for oleclumab with/without durvalumab plus chemotherapy versus chemotherapy alone in the overall population. During dose expansion, an interim analysis indicated that futility criteria in terms of complete and partial responses were not met for arm A2 (oleclumab + GnP) and arm A3 (oleclumab + durvalumab + GnP). Further interim analysis, after data maturity to allow examination of OS and PFS, showed an OS signal and a limited PFS signal in arm A3 versus arm A1, both of which were strongest in the CD73-high subgroup. As there was no improvement in OS or PFS in arm A2 compared with arm A1, arm A2 was terminated and arms A1 and A3 were expanded to reach approximately 70 patients per arm per the study design.

Upon final analysis, there was no statistically significant difference in ORR, PFS, and OS among arms A3, A2, and A1 in the overall population, and numerically worse outcomes observed in arm A2 compared with arm A1 were not statistically significant and were likely the result of the small number of patients in arm A2. These results are generally consistent with the phase II Canadian Cancer Trials Group PA.7 trial comparing GnP with GnP plus durvalumab and tremelimumab as initial therapy in patients with mPDAC, in which the primary endpoint of clinical benefit was not met (20). Although the PA.7 trial did not evaluate CD73 inhibition, it demonstrated that combination chemotherapy with dual checkpoint inhibition did not provide clinical benefit in mPDAC versus combination chemotherapy alone (20). In a phase I trial of quemliclustat (small-molecule CD73 inhibitor) plus GnP in patients with treatment-naïve mPDAC, the addition of zimberelimab (PD1 inhibitor) did not seem to improve outcomes: confirmed ORR (38% vs. 25%), median PFS (8.8 vs. 4.9 months), and median OS (19.4 vs. 14.6 months; ref. 21). Encouraging results have been observed with adenosine pathway blockade in combination with immune checkpoint inhibitors or chemotherapy in patients with other solid tumors (22–24).

In the current study, the prognosis for patients with high CD73 levels in terms of ORR, PFS, and OS was worse than those with low CD73 levels in all treatment arms, with the exception of ORR in arm 2. PFS and OS were improved in patients with high CD73 in arm A3 versus A1; however, this should be interpreted with caution given that these are a subset of patients and that those with high CD73 levels in the control arm (arm A1) in the first interim analysis had poorer results than expected (and were deemed to not be representative of the general PDAC population). The PFS and OS benefit initially seen in arm A3 versus arm A1 in patients enrolled prior to the first interim analysis was not maintained in the patients enrolled after the analysis. The need for a biomarker to identify the patient population likely to benefit from targeting CD73 or the broader adenosine pathway is critical to further the clinical research targeting this immunosuppressive mechanism. In addition, the potential for oleclumab to bind to CD73 on the surface of normal cells may result in insufficient accumulation at the site of the tumor (25).

The ORR in arm A1 (29.0%) was slightly greater than the assumption for standard of care used for sample sizing (23.0%), which was based on historical data (20, 26). This higher than expected ORR in arm A1 did not affect overall interpretation of the results given the lack of efficacy effect seen between arms A3 and A1 and is consistent with a more recently published ORR of 36.2% (27). Any differences in baseline characteristics between treatment groups did not affect the interpretation of results.

### Conclusions

The safety profile of oleclumab plus durvalumab and chemotherapy was consistent with the known safety profiles of the individual components and was manageable. The study did not meet its primary efficacy endpoint of an increase in ORR, and no meaningful improvement was observed in the overall population in PFS and OS for oleclumab with/without durvalumab plus chemotherapy versus chemotherapy alone. Both PFS and OS were improved in patients with high CD73 who received oleclumab plus durvalumab and GnP versus GnP alone, although this should be interpreted with caution given the underperformance of the control arm.

## Supplementary Material

Supplementary Methods S1 Supplementary Methods

Supplementary Results S1 Supplementary Results

Supplementary Figure S1 Study design: dose-escalation and dose-expansion phases

Supplementary Figure S2 Exploratory overall survival analysis of oleclumab + durvalumab + GnP (Arm A3) versus GnP alone (Arm A1) by IHC or ctDNA-based biomarkers per all patients (A) or CD73 high only population (B)

Supplementary Figure S3 ctDNA-derived genomic analysis of tumors from patients with plasma at baseline and IHC biomarkers from the dose-expansion phase of Cohort A

Supplementary Figure S4 CD73 expression by KRAS mutation status (A) and OS and PFS stratified by KRAS mutation status (B)

Supplementary Figure S5 Baseline median variant allelic frequency (VAF) by KRAS status (A), exploratory OS and PFS stratified by baseline median VAF (B) and distribution of KRAS mutation status (C), VAF (D) and blood TMB (E) by arm

Supplementary Table S1 Patient demographics and disease characteristics (dose-escalation phase, as-treated population; N = 25)

Supplementary Table S2 Treatment-emergent adverse events occurring at any severity in ≥20% of patients in any arm in the dose-escalation phase (as-treated population; N = 25)
