# Supplementary material for: A Phase Ib/II Randomized Clinical Trial of Oleclumab with or without Durvalumab plus Chemotherapy in Patients with Metastatic Pancreatic Ductal Adenocarcinoma
Source: Clin Cancer Res. 2024 Aug 6;30(20):4609–17. doi: 10.1158/1078-0432.CCR-24-0499 (PMC11474165; doi:10.1158/1078-0432.CCR-24-0499)
Supplement: Supplementary Methods S1 — Supplementary Methods [file ccr-24-0499_supplementary_methods_s1_suppms1.pdf]

A Phase 1b/2 Randomized Clinical Trial of Oleclumab with or without Durvalumab plus Chemotherapy in Patients with Metastatic Pancreatic Ductal Adenocarcinoma

Andrew L. Coveler, et al.

Supplementary Methods

Objectives and endpoints for dose-escalation phase

The primary objective of the dose-escalation phase was to assess safety and tolerability. Primary endpoints were to determine the recommended Phase 2 dose (RP2D) of oleclumab based on dose-limiting toxicities (DLTs); to assess the incidence of adverse events (AEs) and serious AEs; and to determine clinically meaningful changes from baseline in laboratory parameters and vital signs. Secondary objectives were to characterize preliminary antitumor activity and to evaluate immunogenicity and pharmacokinetics (PK). Secondary endpoints included investigator-assessed objective response rate (ORR) and disease control rate (DCR; defined as complete response, partial response, or stable disease ≥8 weeks) per Response Evaluation Criteria in Solid Tumors (RECIST) v1.1.

Additional inclusion criteria

Patients were required to have adequate hematologic, renal, and hepatic function. Adequate hematologic function was defined as an absolute neutrophil count ≥ 1500 μ/L, a platelet count ≥100,000 μ/L, and hemoglobin ≥9.0 g/dL, all without growth factor or transfusion support within 14 days prior to screening.

Patients had to have ≥1 lesion that was measurable using RECIST v1.1 guidelines. A previously irradiated lesion could be considered a target lesion if it was well defined, measurable per RECIST, and had clearly progressed; patients undergoing tumor biopsies must have had additional non-target lesions that could be biopsied at acceptable risk as judged by the investigator, or if no other lesion was suitable for biopsy then the RECIST target lesion used for biopsy must have been ≥2 cm in longest diameter.

Additional exclusion criteria

Patients were ineligible if they received any conventional or investigational anticancer therapy within 21 days or palliative radiotherapy within 14 days prior to the scheduled first dose of study treatment; had active or prior autoimmune disorders within the past 3 years; or had leptomeningeal disease, or cord compression.

Patients with untreated central nervous system (CNS) metastases were excluded. Those who had been previously treated for CNS metastases that were radiographically and neurologically stable for at least 28 days and did not require corticosteroids for symptomatic management for at least 14 days prior to the scheduled first dose of study treatment were eligible. Patients with current or prior use of immunosuppressive medication within 14 days prior to the scheduled first dose of study treatment were excluded. Patients were also excluded if they had received any live, attenuated vaccine within 28 days before the first dose of study treatment.

Dose-limiting toxicities

DLTs were evaluated during dose-escalation for both Cohorts A and B from the first dose of all study treatments through Day 28. Toxicities that were clearly and directly related to the primary disease, chemotherapy alone, or to another etiology were not considered DLTs. DLTs were defined as any of the following events during the evaluation period:

- Immune-mediated adverse events (imAEs)
    - Any Grade 4 imAE (excluding asymptomatic lipase and/or amylase elevation)
    - Any Grade ≥3 colitis
    - Any Grade ≥3 nausea, vomiting, or diarrhea that does not resolve to Grade 2 or less within 3 days of the initiation of maximal supportive care
    - Any Grade ≥3 pneumonitis or interstitial lung disease (ILD)
    - Any Grade 2 pneumonitis or ILD for which the symptomatology does not resolve within 7 days of the initiation of maximal supportive care

- Anemia
    - Grade 4 anemia of any duration
    - Grade 3 anemia if associated with clinical sequelae or requiring transfusion of >2 units of red blood cells
- Thrombocytopenia
    - Grade 4 thrombocytopenia ≥7 days
    - Grade 3 or 4 thrombocytopenia, regardless of duration, associated with Grade 3 or higher hemorrhage
- Neutropenia and/or febrile neutropenia
    - Grade 4 febrile neutropenia of any duration
    - Grade 3 febrile neutropenia lasting ≥5 days while receiving maximal supportive care
    - Grade 4 neutropenia lasting >7 days
- Liver function tests
    - Isolated Grade 3 liver transaminase elevation or isolated Grade 3 total bilirubin (TBL) elevation that does not downgrade to Grade 1 or less within 14 days after onset with optimal medical management, including systemic corticosteroids
    - Isolated Grade 4 liver transaminase elevation or TBL elevation regardless of duration
    - Any increase in aspartate aminotransferase or alanine aminotransferase (ALT) >3 × upper limit normal (ULN) and concurrent increase in TBL >2 × ULN (Hy's Law) without evidence of cholestasis or alternative explanations (e.g., viral hepatitis, disease progression in the liver)
- Any other toxicity that is greater than that at baseline, is clinically significant and/or unacceptable and is judged to be a DLT by the dose-escalation committee.

The following were not included within the definition of DLT:

- Grade 3 endocrine disorder (thyroid, pituitary, and/or adrenal insufficiency) that was managed with or without systemic corticosteroid therapy and/or hormone replacement therapy
- Grade 3 inflammatory reaction attributed to a local antitumor response (e.g., inflammatory reaction at sites of metastatic disease, lymph nodes, etc.) that resolved to Grade 1 or less within 30 days
- Concurrent vitiligo or alopecia of any AE grade
- Isolated laboratory changes of any grade without clinical sequelae or clinical significance, other than those defined as a DLT above.

Disease assessments

Disease assessments per RECIST v1.1 were performed at screening, and then either every 8 weeks for 48 weeks then every 12 weeks (Cohort A), or every 6 weeks for 24 weeks then every 8 weeks (Cohort B) until confirmed radiological progression in both the dose-escalation and dose-expansion phases.

Biomarker methods

Immunohistochemistry

Screening tumor specimens were obtained and a pathologist examined a hematoxylin and eosin stained slide from each tissue block for the presence of viable tumor. Sections of 4 µm thickness were cut from a representative tumor block selected from each patient for immunohistochemistry (IHC) analysis. The IHC tests used for each biomarker were as follows, using previously optimized Ventana antibody clone SP263 conditions. PD-L1 expression was tested using the IHC assay on the Ventana BenchMark Auto-stainer (Roche Diagnostics, Ventana Medical Systems; Tucson, AZ). CD73 IHC was performed and validated according to College of American Pathologists (CAP) guidelines in a CAP-accredited, Clinical Laboratory Improvement Amendments-certified laboratory with rabbit monoclonal antibody clone EPR6115 (Abcam, #ab124725) at Discovery Lifesciences (formerly

Qualtek; Vista, CA) and scored manually by a pathologist for percentage of positive tumor cells with CD73 membrane expression at 1+/2+/3+ intensities as well as for primary membrane expression pattern: apical/luminal restricted versus complete circumferential. CD39 IHC was performed with rabbit monoclonal antibody clone EPR20461 (Abcam, #ab223843) on the Leica bond platform (Leica Biosystems; Wetzlar, Germany). CD8-purple (Dako, clone M7103) and FoxP3-DAB (Abcam #ab99963, clone SP97) IHC was performed using a 3-plex chromogenic IHC assay with Pan Cytokeratin-yellow (clone AE1/AE3/PCK26, Ventana 760-2135) for automated quantification of immune cells in the epithelial versus the stromal compartment of the tumor microenvironment. All the IHC-stained slides were converted into high resolution digital images of the whole section (e-slide) using Aperio AT Turbo or Aperio XT scanners (Leica Biosystems; Buffalo Grove, IL) with a 20x objective magnification. Digital images were manually annotated by a pathologist to designate the tumor regions for analysis. Marker quantification was performed by AstraZeneca Computational Pathology (formerly Definiens AG) in Munich (Germany) via image analysis using Definiens Developer™ software, employing customized algorithms for CD39 and CD8/FoxP3/PanCK, which were reported as percentage of area with positive CD39 staining in the tumor center or marker-positive cells/mm^2 of tumor area for the 3-plex assay. PD-L1 was scored manually by a pathologist for percentage of positive tumor cells with membrane staining. The numbers of patients with evaluable samples for each marker were: PD-L1 (n=153), CD73 (n=170), CD39 (n=151), and CD8/FoxP3/PanCK (n=120).

Analyses of next generation sequencing (NGS) data from circulating tumor (ct)DNA

NGS data was analyzed using a Guardant ctDNA analysis pipeline (Guardant Health, Redwood City, CA, USA), which starts from the raw sequencing data (binary base call; BCL files) and outputs the final mutation calls. Briefly, the pipeline first performs adapter trimming, barcode checking, and correction. Cleaned paired FASTQ files are aligned to human reference genome build hg19 using the Burrows-Wheeler alignment (BWA) tool. Consensus binary alignment map (BAM) files are then derived by merging paired-end reads originated from the same molecules (based on mapping location and unique molecular identifiers) as single-strand fragments. Single-strand fragments from

the same double-strand DNA molecules were further merged as double-stranded. By using the error suppression method described by Newman and colleagues (1) both sequencing and polymerase chain reaction errors were mostly corrected during this process.

Candidate variants were called by comparing with local variant background (defined based on plasma samples from health donors and historical data). Variants were further filtered by log-odds threshold, base and mapping quality thresholds, repeat regions, and other quality metrics.

Homologous recombination repair-related gene mutations status defined as a mutation in one of the following genes – *BRCA2, ATM, RAD51B, RAD51C, RAD54L, RAD51D, BRIP1, FANCI, FANCL, PALB2, BARD1, CHEK1, CHEK2,* or *CDK12* – as this subgroup defines one of the populations eligible for olaparib based on its ability to enrich for clinical activity with olaparib in the context of the PROfound study (2). The numbers of patients with evaluable baseline ctDNA samples from the dose-expansion phase of Cohort A was n=160.

Statistical considerations

Sample size

The study was planned to recruit approximately 339 patients overall, with 24 patients in the dose-escalation phase (9–12 patients per cohort) and 315 patients in the dose-expansion phase (approximately 70 and 35 patients in each treatment arm of Cohorts A and B, respectively). The sample size of 70 patients per treatment arm in dose-expansion Cohort A was based on an ORR with gemcitabine and nab-paclitaxel (GnP; Arm A1) of 23% and an assumed drop-out rate of 20% providing 78% power at a 1-sided significance level of 0.10 to detect a difference in ORR of 20% (i.e., ORR = 43% in the oleclumab-containing arms [Arms A2 or A3]). The sample size of 35 patients per treatment arm in dose-expansion Cohort B was based upon an ORR with modified (m)FOLFOX (Arm B1) of 7% and an assumed drop-out rate of 10%, providing 72% power at a 1-sided significance level of 0.10 to detect a difference in ORR of 20% (i.e., ORR = 27% in the oleclumab-containing arms [Arms B2 or B3]).

Analysis populations

Population	Description
Intent-to-treat (ITT) population	All patients who were randomized and received any amount of investigational product and were analyzed according to randomized treatment assignment. All analyses were performed on the ITT population unless otherwise specified.
As-treated population	All patients who received any investigational product and were analyzed according to treatment received.
Response-evaluable population	Patients from the as-treated population who had a baseline disease assessment (DA), had the opportunity to be followed for at least 16 (Cohort A) or 12 weeks (Cohort B) at the time of the data cut off and either had at least one post-baseline DA and/or discontinued treatment due to death or disease progression
DLT-evaluable population	All patients enrolled in the dose-escalation phase who received all planned doses of oleclumab, durvalumab, and chemotherapy during the DLT evaluation period (from the first dose of all study treatments through Day 28) and completed the safety follow-up through the DLT evaluation period or experienced any DLT.
PK-evaluable population	All patients who received at least one dose of each investigational product with at least one reportable PK concentration.
Anti-drug antibody (ADA)-evaluable population	All patients in the as-treated population who had a non-missing baseline ADA result and at least one non-missing post-baseline ADA result.

Interim analysis futility criteria

- An interim analysis was performed when approximately 30 patients in each treatment arm of Cohort A dose expansion had been dosed and reached the data cut off criteria (i.e., patients who had a baseline disease assessment, had been dosed at least 16 weeks prior to the time of the data cut-off, and had at least one post-baseline disease assessment and/or discontinued treatment due to death or disease progression).
- Interim analysis compared the difference in ORR (confirmed/unconfirmed complete response or partial response per RECIST v1.1), between treatment arms using a Bayesian predictive probability model applied to the response-evaluable population.
- The interim futility criteria were derived in three steps:
    - The target value was set to demonstrate a 20% increase in the experimental arm ORR over the control.
    - Final No-Go criteria upon full randomization were met if the probability that the true difference of the ORR between experimental and control arms exceeded the pre-specified target value of 20% was less than 10%.
    - Interim futility criteria were met if it predicted that there was a high probability of reaching a final No-Go decision upon full randomization of planned patients given the existing observed data (i.e., predictive probability of a final No-Go decision >90%).

References

1. Newman AM, Lovejoy AF, Klass DM, Kurtz DM, Chabon JJ, Scherer F, et al. Integrated digital error suppression for improved detection of circulating tumor DNA. Nat Biotechnol. 2016 May;34(5):547–555. doi: 10.1038/nbt.3520.

2. de Bono J, Mateo J, Fizazi K, Saad F, Shore N, Sandhu S, et al. Olaparib for Metastatic Castration-Resistant Prostate Cancer. N Engl J Med. 2020 May 28;382(22):2091–2102. doi: 10.1056/NEJMoa1911440.
