# Supplementary material for: A Phase Ib/II Randomized Clinical Trial of Oleclumab with or without Durvalumab plus Chemotherapy in Patients with Metastatic Pancreatic Ductal Adenocarcinoma
Source: Clin Cancer Res. 2024 Aug 6;30(20):4609–17. doi: 10.1158/1078-0432.CCR-24-0499 (PMC11474165; doi:10.1158/1078-0432.CCR-24-0499)
Supplement: Supplementary Results S1 — Supplementary Results [file ccr-24-0499_supplementary_results_s1_supprs1.pdf]

A Phase 1b/2 Randomized Clinical Trial of Oleclumab with or without Durvalumab plus Chemotherapy in Patients with Metastatic Pancreatic Ductal Adenocarcinoma

Andrew L. Coveler, et al.

Supplementary Results

Dose-escalation phase

Patient demographics and disease characteristics

Patient demographics and baseline disease characteristics were generally comparable among cohorts and treatment groups, except for the median CA19-9 level and the proportion of patients with an Eastern Cooperative Oncology Group performance score of 1, which were both higher in patients receiving oleclumab 1500 mg in Cohort A than in the other treatment groups (**Supplementary Table 1**).

Fourteen patients received first-line treatment in Cohort A (n=7 each for oleclumab 1500 mg and 3000 mg) and 11 patients received second-line treatment in Cohort B (oleclumab 1500 mg, n=3; oleclumab 3000 mg, n=8). All patients in both cohorts discontinued chemotherapy and immunotherapy, most commonly due to progressive disease, and had died by the end of the study.

Dose-limiting toxicities

The DLT-evaluable population in Cohort A consisted of five patients who received oleclumab 1500 mg and six patients who received oleclumab 3000 mg. There were no DLTs in Cohort A. The DLT-evaluable population in Cohort B included three patients who received oleclumab 1500 mg and eight patients who received oleclumab 3000 mg. One patient (12.5%) from Cohort B who received oleclumab 3000 mg experienced two DLTs of Grade 3 localized edema (attributed to oleclumab) and Grade 3 nausea (attributed to mFOLFOX). Oleclumab 3000 mg was the protocol-specified maximum administered dose and was chosen as the RP2D for dose-expansion Cohort A; enrollment into dose-expansion Cohort B was not opened.

Safety

The median number of oleclumab doses in the dose-escalation phase was four in Cohort A (range 1–12) and five in Cohort B (range 2–15). Overall safety profiles were generally similar in Cohorts A and B (**Supplementary Table 2**) and consistent with the known individual safety profiles of oleclumab, durvalumab, and GnP. Most common treatment-emergent AEs (TEAEs) (occurring in ≥50% of patients) in Cohort A were fatigue (92.9%), nausea (85.7%), and ALT increased, constipation, decreased appetite, and diarrhea (each, 50.0%). Most common TEAEs (occurring in ≥50% of patients) in Cohort B were fatigue (72.7%), nausea (63.6%), and diarrhea (54.5%).

TEAEs of special interest (AESI) for oleclumab occurred in nine patients (64.3%) in Cohort A and five patients (45.5%) in Cohort B. AESI for durvalumab occurred in 14 patients (100%) in Cohort A and nine patients (81.8%) in Cohort B. AESI categories for oleclumab and durvalumab across the Cohorts are listed in **Supplementary Table 3**.

Three patients (21.4%) in Cohort A and three patients (27.3%) in Cohort B discontinued treatment permanently due to AEs. One patient in Cohort A receiving oleclumab 1500 mg died due to a TEAE (pneumonia, not treatment-related) and none died due to TEAEs in Cohort B.

Grade ≥3 AEs related to one or more treatments (treatment-related AEs; TRAEs) were reported in six patients (42.9%) in Cohort A and seven patients (63.6%) in Cohort B. The most common Grade ≥3 TRAEs were hematologic events (n=5), and gastrointestinal events and investigations (each, n=3) in Cohort A, and investigations (n=4), and general disorders and hematologic events in Cohort B. Serious TRAEs were reported in three patients (21.4%) in Cohort A (vomiting [n=2], pyrexia, acute kidney injury, and deep vein thrombosis [n=1, each] and one patient (9.1%) in Cohort B (nausea and localized edema); all at oleclumab 3000 mg. There were no deaths due to treatment-related toxicity.

Pharmacokinetics and immunogenicity

During dose-escalation, oleclumab serum concentrations increased with increasing doses (**Supplementary Table 7**).

The immunogenicity of oleclumab was evaluable in 23 patients. None had ADAs at baseline and two patients had ADAs post baseline.

Efficacy

One patient in Cohort A who received oleclumab 3000 mg had a confirmed partial response. The ORR for this dose was 14.3% (95% confidence interval [CI]: 0.4%, 57.9%). Seven patients in Cohort A had stable disease, including two with unconfirmed partial responses. DCR was 42.9% (n=3) in patients receiving oleclumab 1500 mg and 71.4% (n=5) in patients receiving oleclumab 3000 mg. One patient in Cohort B receiving oleclumab 3000 mg had a confirmed partial response. The ORR for this dose was 12.5% (95% CI: 0.3, 52.7). Six patients in Cohort B had stable disease, including two with unconfirmed partial responses. DCR was 66.7% (n=2) in patients who received oleclumab 1500 mg and 62.5% (n=5) in patients who received oleclumab 3000 mg.
