# Supplementary material for: A Phase Ib/II Randomized Clinical Trial of Oleclumab with or without Durvalumab plus Chemotherapy in Patients with Metastatic Pancreatic Ductal Adenocarcinoma
Source: Clin Cancer Res. 2024 Aug 6;30(20):4609–17. doi: 10.1158/1078-0432.CCR-24-0499 (PMC11474165; doi:10.1158/1078-0432.CCR-24-0499)
Supplement: Supplementary Figure S1 — Study design: dose-escalation and dose-expansion phases [file ccr-24-0499_supplementary_figure_s1_suppfs1.pdf]

Supplementary Figure 1. Study design: dose-escalation and dose-expansion phases

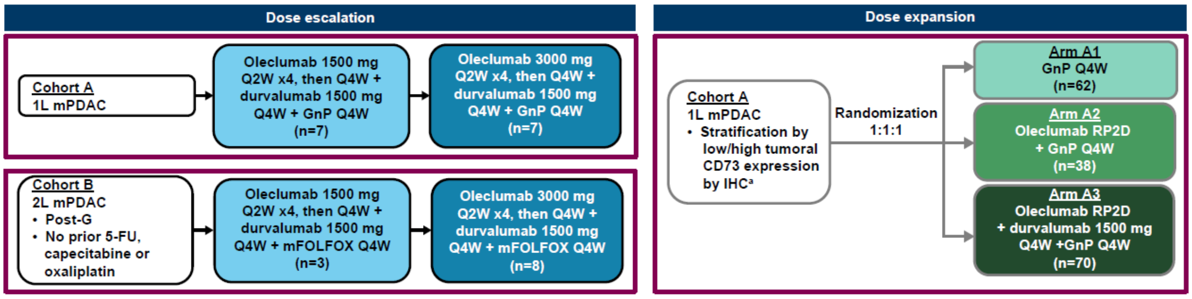

[a]CD73 low: CD73 expression of 2+ or 3+ intensity in <50% of tumor cells; CD73 high: CD73 expression of 2+ or 3+ intensity in ≥50% of tumor cells

1/2L, first/second line; 5-FU, 5-fluorouracil; G, gemcitabine; GnP, gemcitabine and nab-paclitaxel; mFOLFOX, modified regimen of leucovorin, 5-FU, and oxaliplatin; mPDAC, metastatic pancreatic ductal adenocarcinoma; Q2W, every 2 weeks; Q4W, every 4 weeks; RP2D, recommended Phase 2 dose.
