# Supplementary material for: A Phase Ib/II Randomized Clinical Trial of Oleclumab with or without Durvalumab plus Chemotherapy in Patients with Metastatic Pancreatic Ductal Adenocarcinoma
Source: Clin Cancer Res. 2024 Aug 6;30(20):4609–17. doi: 10.1158/1078-0432.CCR-24-0499 (PMC11474165; doi:10.1158/1078-0432.CCR-24-0499)
Supplement: Supplementary Figure S2 — Exploratory overall survival analysis of oleclumab + durvalumab + GnP (Arm A3) versus GnP alone (Arm A1) by IHC or ctDNA-based biomarkers per all patients (A) or CD73 high only population (B) [file ccr-24-0499_supplementary_figure_s2_suppfs2.pdf]

Supplementary Figure 2. Exploratory overall survival analysis of oleclumab + durvalumab + GnP (Arm A3) versus GnP alone (Arm A1) by IHC or ctDNA-based biomarkers per all patients (A) or CD73 high only population (B)

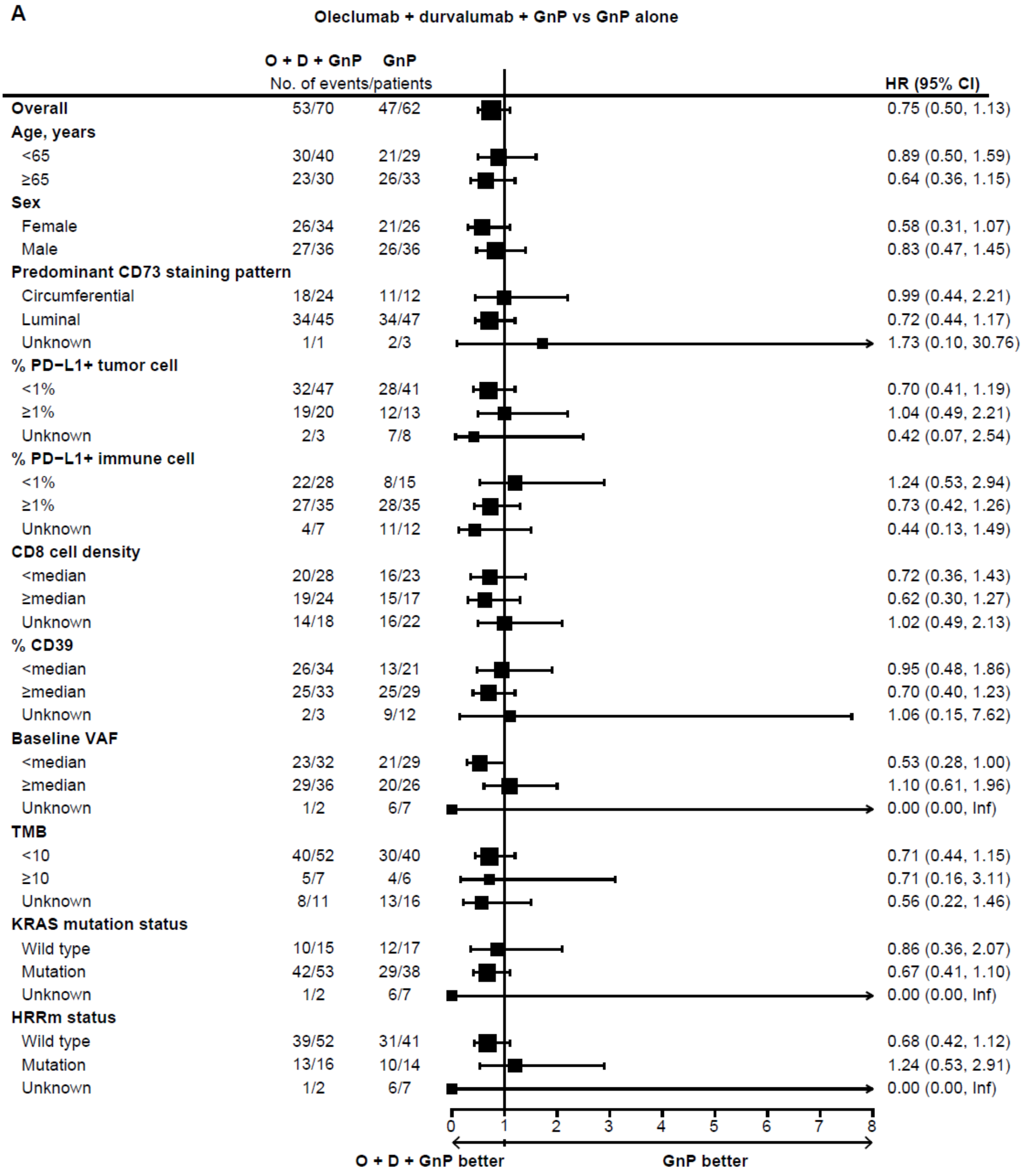

B

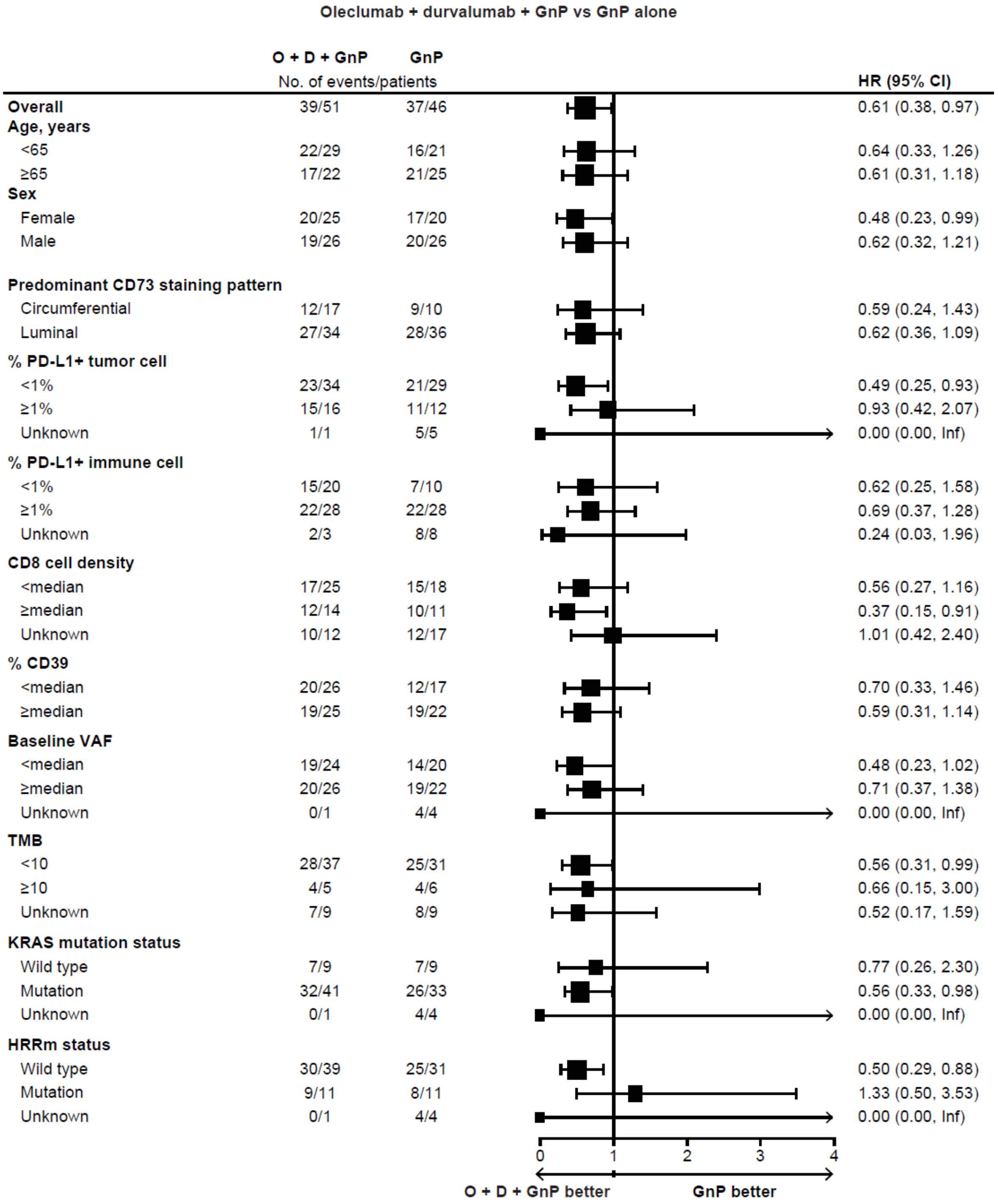

HR calculations were performed using Cox regression models in all patients or those with high CD73 expression.

CD, cluster of differentiation; CI, confidence interval; D, durvalumab; GnP, gemcitabine and nab-paclitaxel; HR, hazard ratio; HRRm, homologous recombination repair-related gene mutation; Inf,

infinity; O, oleclumab; PD-L1, programmed death-ligand 1; TMB, tumor mutational burden; VAF, variant allelic frequency.
