# Supplementary material for: A Phase Ib/II Randomized Clinical Trial of Oleclumab with or without Durvalumab plus Chemotherapy in Patients with Metastatic Pancreatic Ductal Adenocarcinoma
Source: Clin Cancer Res. 2024 Aug 6;30(20):4609–17. doi: 10.1158/1078-0432.CCR-24-0499 (PMC11474165; doi:10.1158/1078-0432.CCR-24-0499)
Supplement: Supplementary Figure S3 — ctDNA-derived genomic analysis of tumors from patients with plasma at baseline and IHC biomarkers from the dose-expansion phase of Cohort A [file ccr-24-0499_supplementary_figure_s3_suppfs3.pdf]

Supplementary Figure 3. ctDNA-derived genomic analysis of tumors from patients with plasma at baseline and IHC biomarkers from the dose-expansion phase of Cohort A

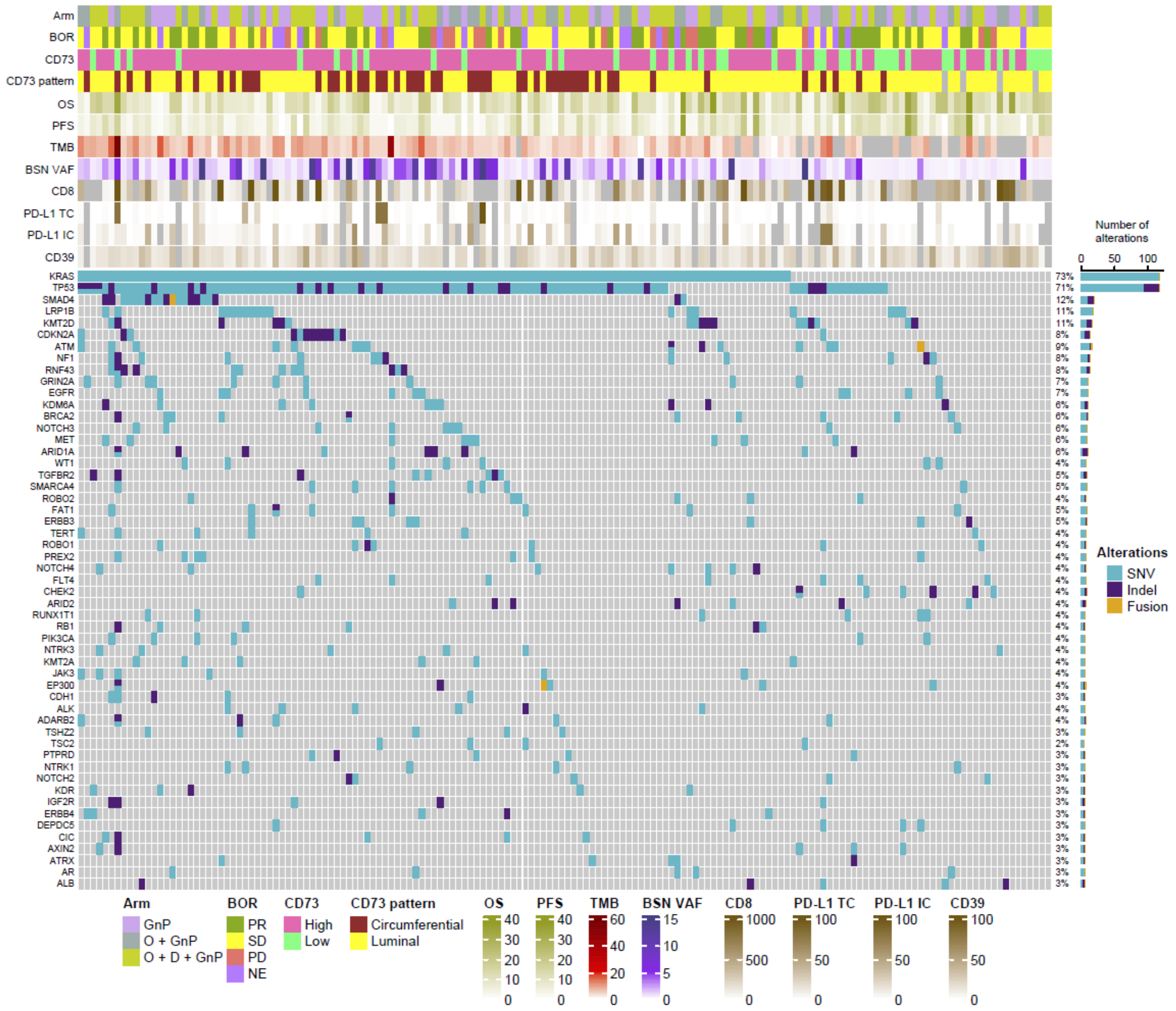

BOR, best objective response; BSN VAF, baseline median variant allelic frequency; CD, cluster of differentiation; ctDNA, circulating tumor DNA; D, durvalumab; GnP, gemcitabine + nab-paclitaxel; IC, immune cell; IHC, immunohistochemistry; Indel, insertion-deletion mutation; NE, not estimable; O, oleclumab; OS, overall survival (months); PD, progressive disease; PD-L1, programmed death-ligand 1; PFS, progression-free survival (months); PR, partial response; SD, stable disease; SNV, single nucleotide variant; TC, tumor cell; TMB, tumor mutational burden.
