# Supplementary material for: A Phase Ib/II Randomized Clinical Trial of Oleclumab with or without Durvalumab plus Chemotherapy in Patients with Metastatic Pancreatic Ductal Adenocarcinoma
Source: Clin Cancer Res. 2024 Aug 6;30(20):4609–17. doi: 10.1158/1078-0432.CCR-24-0499 (PMC11474165; doi:10.1158/1078-0432.CCR-24-0499)
Supplement: Supplementary Figure S4 — CD73 expression by KRAS mutation status (A) and OS and PFS stratified by KRAS mutation status (B) [file ccr-24-0499_supplementary_figure_s4_suppfs4.pdf]

Supplementary Figure 4. CD73 expression by *KRAS* mutation status (A) and OS and PFS stratified by *KRAS* mutation status (B)

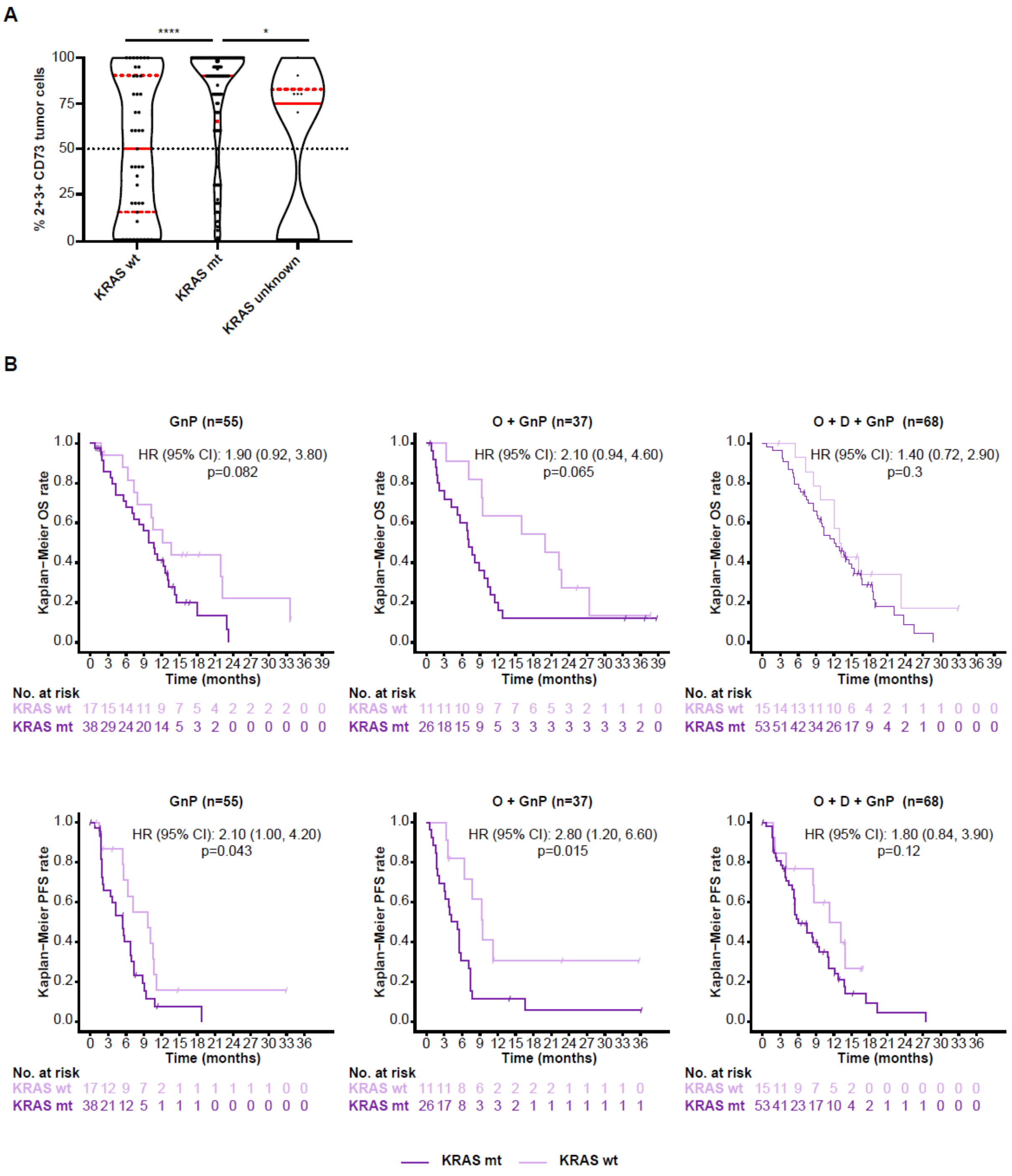

*p<0.05; ****p<0.0001

CD, cluster of differentiation; CI, confidence interval; D, durvalumab; GnP, gemcitabine + nab-paclitaxel; HR, hazard ratio; mt, mutation; O, oleclumab; OS, overall survival; PFS, progression-free survival; wt, wild type.
