# Supplementary material for: A Phase Ib/II Randomized Clinical Trial of Oleclumab with or without Durvalumab plus Chemotherapy in Patients with Metastatic Pancreatic Ductal Adenocarcinoma
Source: Clin Cancer Res. 2024 Aug 6;30(20):4609–17. doi: 10.1158/1078-0432.CCR-24-0499 (PMC11474165; doi:10.1158/1078-0432.CCR-24-0499)
Supplement: Supplementary Figure S5 — Baseline median variant allelic frequency (VAF) by KRAS status (A), exploratory OS and PFS stratified by baseline median VAF (B) and distribution of KRAS mutation status (C), VAF (D) and blood TMB (E) by arm [file ccr-24-0499_supplementary_figure_s5_suppfs5.pdf]

Supplementary Figure 5. Baseline median variant allelic frequency (VAF) by *KRAS* status (A), exploratory OS and PFS stratified by baseline median VAF (B) and distribution of *KRAS* mutation status (C), VAF (D) and blood TMB (E) by arm

A

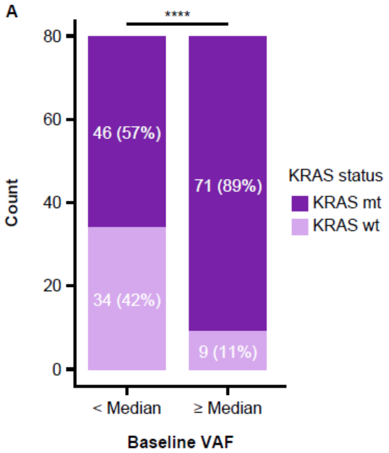

B

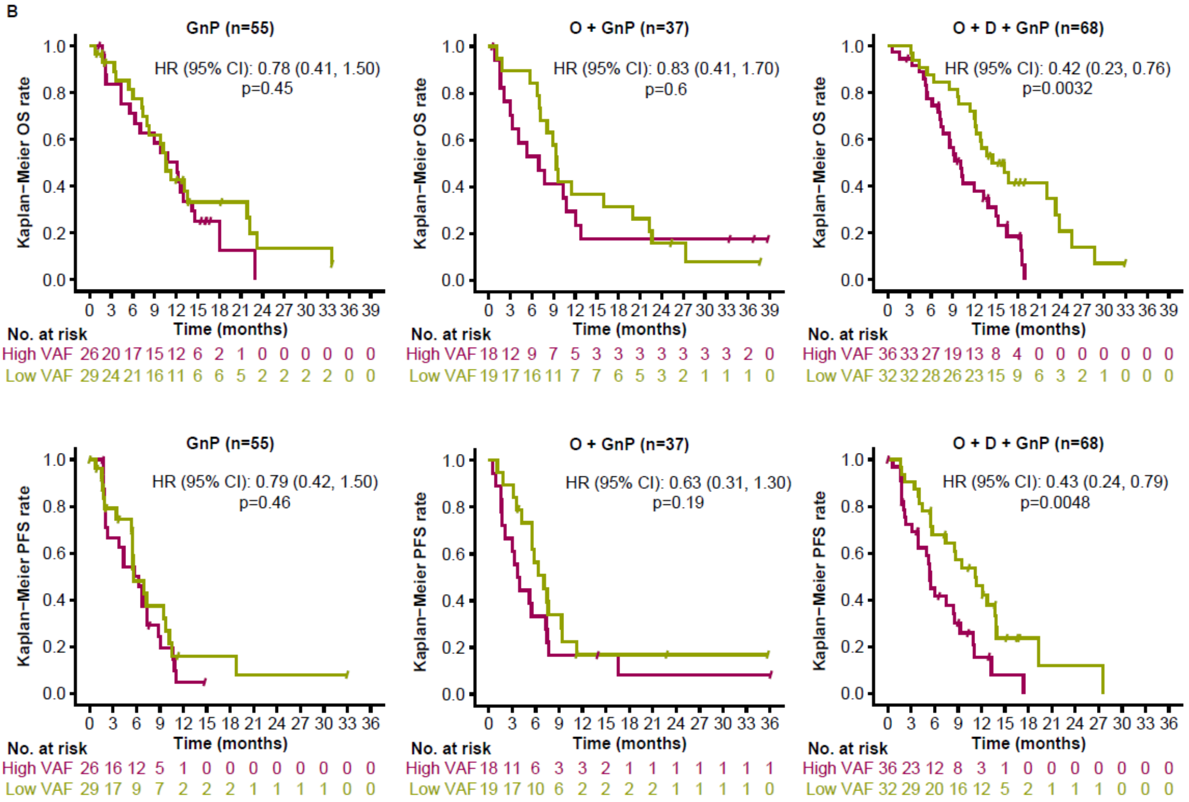

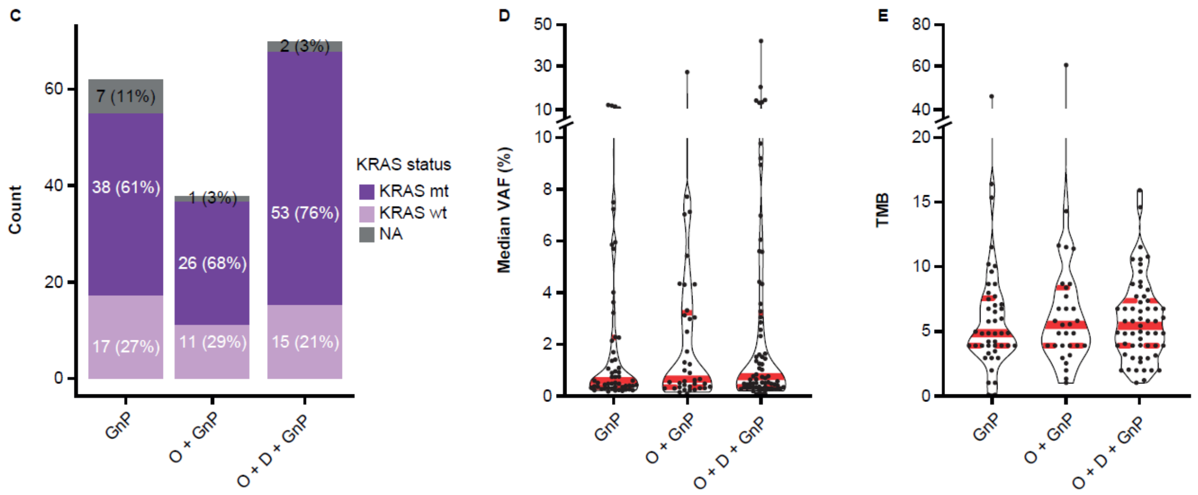

****p<0.0001

BSN, baseline; CI, confidence interval; D, durvalumab; GnP, gemcitabine + nab-paclitaxel; grp, group; HR, hazard ratio; mut, mutation; O, oleclumab; OS, overall survival; PFS, progression-free survival; TMB, tumor mutational burden; VAF, variant allelic frequency; wt, wild type.
