# Supplementary material for: A Phase Ib/II Randomized Clinical Trial of Oleclumab with or without Durvalumab plus Chemotherapy in Patients with Metastatic Pancreatic Ductal Adenocarcinoma
Source: Clin Cancer Res. 2024 Aug 6;30(20):4609–17. doi: 10.1158/1078-0432.CCR-24-0499 (PMC11474165; doi:10.1158/1078-0432.CCR-24-0499)
Supplement: Supplementary Table S1 — Patient demographics and disease characteristics (dose-escalation phase, as-treated population; N = 25) [file ccr-24-0499_supplementary_table_s1_suppts1.pdf]

Supplementary Table 1. Patient demographics and disease characteristics (dose-escalation phase, as-treated population; N=25)

	Dose-escalation phase (N=25)			
	Cohort A		Cohort B	
	**O 1500 mg + D 1500 mg + GnP N=7**	**O 3000 mg + D 1500 mg + GnP N=7**	**O 1500 mg + D 1500 mg + mFOLFOX N=3**	**O 3000 mg + D 1500 mg + mFOLFOX N=8**
Median age (range), years	71.0 (40, 76)	61.0 (41, 70)	73.0 (50, 78)	65.0 (55, 72)
Sex, n (%)				
Female	3 (42.9)	4 (57.1)	1 (33.3)	4 (50.0)
Male	4 (57.1)	3 (42.9)	2 (66.7)	4 (50.0)
Race, n (%)				
American Indian or Alaska Native	0	0	0	0
Asian	0	0	0	0
Black or African American	0	1 (14.3)	0	2 (25.0)

Native Hawaiian or other Pacific Islander	1 (14.3)	0	0	0
White	6 (85.7)	5 (71.4)	3 (100)	6 (75.0)
Other	0	0	0	0
≥2 races	0	1 (14.3)	0	0
ECOG PS, n (%)				
0	0	3 (42.9)	1 (33.3)	3 (37.5)
1	7 (100)	4 (57.1)	2 (66.7)	5 (62.5)
Time since initial diagnosis, median (range), months	0.8 (0, 52)	0.7 (0, 1)	8.5 (6, 13)	9.4 (3, 15)
Baseline CA19-9, median (range), U/mL	4227.5 (61, 127004)	728.0 (7, 23370)	2407.2 (334, 2825)	929.5 (53, 41682)
Liver metastasis, n (%)				
Yes	4 (57.1)	4 (57.1)	2 (66.7)	6 (75.0)
No/Not specified	3 (42.9)	3 (42.9)	1 (33.3)	2 (25.0)

D, durvalumab; ECOG PS, Eastern Cooperative Oncology Group performance status; GnP, gemcitabine + nab-paclitaxel; mFOLFOX, modified regimen of leucovorin, 5-fluorouracil and oxaliplatin; O, oleclumab.
