# Supplementary material for: A Phase Ib/II Randomized Clinical Trial of Oleclumab with or without Durvalumab plus Chemotherapy in Patients with Metastatic Pancreatic Ductal Adenocarcinoma
Source: Clin Cancer Res. 2024 Aug 6;30(20):4609–17. doi: 10.1158/1078-0432.CCR-24-0499 (PMC11474165; doi:10.1158/1078-0432.CCR-24-0499)
Supplement: Supplementary Table S2 — Treatment-emergent adverse events occurring at any severity in ≥20% of patients in any arm in the dose-escalation phase (as-treated population; N = 25) [file ccr-24-0499_supplementary_table_s2_suppts2.pdf]

Supplementary Table 2. Treatment-emergent adverse events occurring at any severity in ≥20% of patients in any arm in the dose-escalation phase (as-treated population; N=25)

**TEAE, n (%)**	**Cohort A, 1L O + D + GnP (N=14)**		**Cohort B, 2L O + D + mFOLFOX (N=11)**	
	**Any Grade**	**≥ Grade 3**	**Any Grade**	**≥ Grade 3**
Any TEAE	14 (100)	10 (71.4)	11 (100)	9 (81.8)
TEAEs occurring in >20% of patients in any cohort[a]				
Fatigue	13 (92.9)	0	8 (72.7)	1 (9.1)
Nausea	12 (85.7)	2 (14.3)	7 (63.6)	2 (18.2)
Diarrhea	7 (50.0)	1 (7.1)	6 (54.5)	0
ALT increased	7 (50.0)	1 (7.1)	2 (18.2)	1 (9.1)
Constipation	7 (50.0)	0	4 (36.4)	1 (9.1)
Decreased appetite	7 (50.0)	0	4 (36.4)	0
Anemia	6 (42.9)	4 (28.6)	1 (9.1)	0

TEAE, n (%)	Cohort A, 1L<br><br>O + D + GnP (N=14)		Cohort B, 2L<br><br>O + D + mFOLFOX<br><br>(N=11)	
	Any Grade	≥ Grade 3	Any Grade	≥ Grade 3
AST increased	6 (42.9)	1 (7.1)	3 (27.3)	1 (9.1)
Dehydration	6 (42.9)	0	3 (27.3)	0
Pyrexia	6 (42.9)	0	1 (9.1)	0
Vomiting	5 (35.7)	2 (14.3)	4 (36.4)	1 (9.1)
Neutrophil count decreased	2 (14.3)	1 (7.1)	4 (36.4)	3 (27.3)
Peripheral edema	5 (35.7)	0	2 (18.2)	0
Abdominal pain	4 (28.6)	1 (7.1)	3 (27.3)	1 (9.1)
Thrombocytopenia	4 (28.6)	0	3 (27.3)	0
Neutropenia	4 (28.6)	1 (7.1)	3 (27.3)	2 (18.2)
Platelet count decreased	4 (28.6)	1 (7.1)	3 (27.3)	0
Arthralgia	4 (28.6)	0	2 (18.2)	0
Peripheral neuropathy	4 (28.6)	0	2 (18.2)	0

TEAE, n (%)	Cohort A, 1L<br><br>O + D + GnP (N=14)		Cohort B, 2L<br><br>O + D + mFOLFOX<br><br>(N=11)	
	**Any Grade**	**≥ Grade 3**	**Any Grade**	**≥ Grade 3**
Asthenia	4 (28.6)	1 (7.1)	0	0
Alopecia	4 (28.6)	0	0	0
Stomatitis	0	0	3 (27.3)	0
Hypokalemia	3 (21.4)	1 (7.1)	2 (18.2)	0
Dysgeusia	3 (21.4)	0	2 (18.2)	0
Blood ALP increased	3 (21.4)	1 (7.1)	1 (9.1)	0
Hypotension	3 (21.4)	0	1 (9.1)	0
Hypomagnesemia	3 (21.4)	0	1 (9.1)	0
Acute kidney injury	3 (21.4)	3 (21.4)	0	0
Blood creatinine increased	3 (21.4)	3 (21.4)	0	0
Pneumonia	3 (21.4)	2 (14.3)	0	0
Cough	3 (21.4)	0	0	0

TEAE, n (%)	Cohort A, 1L<br>O + D + GnP (N=14)		Cohort B, 2L<br>O + D + mFOLFOX<br>(N=11)	
	**Any Grade**	**≥ Grade 3**	**Any Grade**	**≥ Grade 3**
Deep vein thrombosis	3 (21.4)	0	0	0
Sinus congestion	3 (21.4)	0	0	0

[a]TEAEs and ≥ Grade 3 TEAEs listed in order of total any grade frequency in either cohort. Patients are counted once for each System Organ Class and Preferred Term regardless of the number of events.

MedDRA version: 25.0.

ALP, alkaline phosphatase; ALT, alanine aminotransferase; AST, aspartate aminotransferase; D, durvalumab; GnP, gemcitabine + nab-paclitaxel; MedDRA, Medical Dictionary for Regulatory Activities; mFOLFOX, modified regimen of leucovorin, 5-fluorouracil and oxaliplatin; O, oleclumab; TEAE, treatment-emergent adverse event.
